# Mental disorders and discrimination: A prospective cohort study of young twin pairs in Germany

**DOI:** 10.1016/j.ssmph.2024.101622

**Published:** 2024-02-07

**Authors:** Lucas Calais-Ferreira, Gregory Armstrong, Elisabeth Hahn, Giles Newton-Howes, James Foulds, John L. Hopper, Frank M. Spinath, Paul Kurdyak, Jesse T. Young

**Affiliations:** a Centre for Mental Health and Community Wellbeing, Melbourne School of Population and Global Health, The University of Melbourne, Melbourne, Victoria, Australia; b Centre for Adolescent Health, Murdoch Children's Research Institute and Royal Children's Hospital, Melbourne, Victoria, Australia; c Justice Health Group, School of Population Health, Curtin University, Perth, Western Australia, Australia; d Nossal Institute for Global Health, Melbourne School of Population and Global Health, The University of Melbourne, Melbourne, Victoria, Australia; e Department of Psychology, Saarland University, Saarbruecken, Germany; f Department of Psychological Medicine, University of Otago, Wellington, New Zealand; g Department of Psychological Medicine, University of Otago, Christchurch, New Zealand; h Centre for Epidemiology and Biostatistics, Melbourne School of Population and Global Health, The University of Melbourne, Melbourne, Victoria, Australia; i ICES, Toronto, Ontario, Canada; j Department of Psychiatry, Temerty Faculty of Medicine, University of Toronto, Toronto, Ontario, Canada; k Institute of Health Policy, Management and Evaluation, Dalla Lana School of Public Health, Temerty Faculty of Medicine, University of Toronto, Toronto, Ontario, Canada; l Centre for Addiction and Mental Health, Toronto, Ontario, Canada; m School of Population and Global Health, The University of Western Australia, Perth, Western Australia, Australia; n National Drug Research Institute, Curtin University, Perth, Western Australia, Australia

**Keywords:** Stigma, Mental health, Adolescence, Youth, Social determinants of health, Twin study

## Abstract

**Background:**

Mental disorders and perceived discrimination share common risk factors. The association between having a mental disorder and experiencing discrimination is well-known, but the extent to which familial factors, such as genetic and shared environmental factors, might confound this association, including sex differences in familial confounding, remains unexplored.

**Aims:**

We investigated potential unmeasured familial confounding in the association between mental disorders and perceived discrimination using a matched twin study design.

**Method:**

We examined data from 2044 same-sex twin pairs (n = 4088) aged 16–25 years from the German population-based study *‘TwinLife'*. We applied random-effects logistic regression to within-individual and within-and-between pair models of the association between mental disorder and perceived discrimination, and used likelihood ratio tests (LRTs) to compare these models. Multivariable models were adjusted for body mass index, educational attainment, and life satisfaction.

**Results:**

There were 322 (8.1%) participants with a diagnosed mental disorder, and 15% (n = 604) of the cohort reported having experienced discrimination in the previous 12 months. Mental disorder and discrimination were associated in the adjusted within-individual model (adjusted odds ratio = 2.19, 95% confidence interval: 1.42–3.39, *P*<0.001). However, the within-and-between pair model showed that this association was explained by the within-pair mean (aOR = 4.24, 95% CI: 2.17–8.29, *P*<0.001) and not the within-pair difference (aOR = 1.26, 95% CI: 0.70–2.28, *P* = 0.4) of mental disorder. Therefore, this association was mostly explained by familial confounding, which is also supported by the LRTs for the unadjusted and adjusted models (*P*<0.001 and *P* = 0.03, respectively). This familial confounding was more prominent for males than females.

**Conclusions:**

Our findings show that the association between mental disorder and discrimination is at least partially explained by unmeasured familial factors. Designing family-based healthcare models and incorporating family members in interventions targeted at ameliorating mental ill-health and experiences of discrimination among adolescents may improve efficacy.

## Highlights

• For the first time, we investigated the association between mental disorder and discrimination while accounting for shared familial factors.
• Mental disorder and discrimination tends to cluster among people of the same familial background.
• Family-based interventions and models of mental healthcare are more likely to be effective for families affected by racism and discrimination.

## 1 Introduction

Mental disorders and perceived discrimination are part of a cycle that keeps some people in pockets of social disadvantage and exclusion (Thornicroft et al., 2022). Mental disorders are defined as clinically significant disturbances related to mental functioning, and include an array of conditions such as depression, anxiety, and neurodevelopmental and sleep disorders (American Psychiatric Association, 2022). It is estimated that about one in three children and adolescents meet established lifetime criteria for mental disorders in the United States (US), although only a proportion of those are likely to be diagnosed and require intervention (Costello et al., 2003; Merikangas et al., 2009) – these numbers might have increased as a result of the COVID-19 pandemic (Ravens-Sieberer et al., 2022). In Germany, data suggests that around 20% of children and adolescents experience mental health problems (Klipker et al., 2018).

The prevalence of self-reported discrimination (for the last five years) based on ethnic or immigrant background, skin colour, or religion in German adults is nearly 40% (European Union Agency for Fundamental Rights, 2017). Discrimination has been linked to multiple negative social and health outcomes (Fiselier et al., 2022), and the risk of experiencing discrimination can reach 80% for young people (15–25 years) facing multiple sources of social disadvantage (Grollman, 2012).

Discrimination experienced by young people is associated with social determinants of health such as education (Kelaher et al., 2008), family income (Assari & Caldwell, 2018), and ethnic background (Armstrong et al., 2022), that commonly cluster among families. This familial clustering occurs largely because most people are likely to have the same or very similar socioeconomic characteristics to their immediate (first-degree) family members from a young age. Individual differences in mental disorders are also likely to be caused, at least partially, by a combination of familial factors of genetic and environmental (including epigenetic) nature (Goes et al., 2012).

Families of people with a mental disorder are more likely to experience social isolation and economic hardship (Tsang et al., 2003). An earlier onset of mental ill-health has been associated with decreased levels of treatment access and poorer health and social outcomes compared to those who develop symptoms later in life (de Girolamo et al., 2012). However, the extent to which associations between mental disorders and these and other social determinants of health are due to shared familial factors is largely unknown.

Mental disorders and perceived discrimination are known to be positively associated, and there are several candidate (and potentially causal) pathways linking these two factors together; mental disorders might precede discrimination or this pathway to be reversed, and they might also co-occur (Bhui, 2016). Regardless of potential direction and causality in this association, understanding its nature is critical because if it is affected by familial confounding, youth mental health and/or discrimination interventions targeted at the individual level will not produce the desired effect. It is possible to adjust for familial confounding in a given association by studying twin pairs who are, by design, uniquely matched for age, sex (for same-sex twin pairs), and several familial factors such as their shared home environment, household socioeconomic status, and (at least partially) their genetic factors (Craig et al., 2020).

Therefore, using a matched co-twin design in a large cohort of adolescent and young adult twins of both sexes in Germany, we aimed to: (1) investigate the within-individual association between any previous diagnosis of mental disorder (including anxiety, depression, alcohol use disorder, attention-deficit hyperactivity disorder (ADHD), or sleep disorder) and perceived discrimination; (2) determine if and to what extent the association between having any of the above diagnosed mental disorders and perceived discrimination is confounded by shared familial factors; and (3) determine if such familial confounding differs by sex.

## 2 Methods

This was a prospective study following two cohorts of young (16 and 25 years of age on average at baseline) same-sex twin pairs from the *TwinLife* Study (Hahn et al., 2016). The *TwinLife* Study has recruited and collected comprehensive data from twin pairs and their family members in Germany through a combination of in-person visits to families every two years and phone interviews in the intervening years. Our study used data from the first wave (i.e., baseline) conducted in 2014/2015 and the second wave (e.g., follow-up) in 2016/2017, both through face-to-face household visits. A total of 4088 twins from 2044 same-sex twin pairs were included in our study.

### 2.1 Outcome

Our outcome of interest was defined as a recent experience of discrimination obtained through self-report to the question *“During the last* 12 months*, have you felt that you were disadvantaged or discriminated against due to any personal characteristics (e.g., your ethnic or cultural background, gender, religious beliefs)?".* We considered a participant's report of experiencing discrimination in either waves 1 or 2 as positive for perceived discrimination.

### 2.2 Baseline measures

Our primary exposure, a previous diagnosis of a mental disorder, was ascertained through self-report in the baseline survey (wave 1) through the question *“Has a doctor ever diagnosed you with one or more of the following illnesses?".* Participants who responded yes to anxiety disorder, depression, alcohol use disorder, ADHD, or sleep disorder were considered a positive for mental disorder (dichotomised as a yes/no variable). These mental disorders were selected for the analysis because they were the only ones captured in the Twinlife surveys and, therefore, available for our analysis.

Sex was ascertained from the original recruitment process based on community registration offices and was confirmed at the first home visit. Only same-sex twin pairs were originally recruited for the Twinlife Study. Other measures ascertained at baseline included body mass index (BMI), current smoker status (yes/no), and global life satisfaction measured as a compiled score (from 5 to 25) from five different domains. This global life satisfaction measure included participant ratings (from strongly disagree to strongly agree on a 5-point scale) for the following statements: *“In most ways, my life is close to my ideal", “the conditions of my life are excellent", “I am satisfied with my life", “so far I have gotten the important things I want in life", and “if I could live my life over, I would change almost nothing"* (Diener et al., 1985).

Self-report of whether the participant had left school before obtaining a primary or secondary school completion certificate (yes/no) was ascertained from both baseline and follow-up surveys (i.e., wave 1 and 2) such that a positive response in either survey was considered positive for leaving school prematurely. Migrant status was ascertained through the self-reported country of origin. Those who reported being from Germany were coded as 0, and those who reported as being from another country were coded as 1. There were 11 individual twins with missing data for migrant status, none of them from the same pair; in these cases, we assigned migrant status based on the twin with available data. Age was matched between the twin pairs and was ascertained at baseline.

### 2.3 Statistical analyses

Descriptive statistics were calculated for all measures. We used Chi-squared tests and non-parametric Wilcoxon rank-sum tests to assess differences between groups for binary and continuous variables, respectively. We calculated sex-adjusted intra-class correlations (ICC) within twin pairs for all covariates and a sex-adjusted familial risk ratio (FRR) for mental disorder.

In our regression analysis, which included monozygotic (MZ) and dizygotic (DZ) pairs together, we first fitted the within-individual models to study the univariable and multivariable associations between mental disorder and perceived discrimination. Second, we fitted the within-and-between pair models to study the same associations, with the addition of adjusting for unmeasured familial confounding, such as genetic and shared environmental factors.

To study the within-individual association of mental disorder and perceived discrimination (aim 1), we fitted univariable and multivariable logistic regression models with random effects applying maximum likelihood estimation of odds ratios (OR). This allowed us to study within-individual associations between exposure and outcome (including covariates) while accounting for the paired structure of the data to make inferences about individual differences. Our multivariable models were adjusted for sex and two other risk factors with previous evidence of an association with mental health and perceived discrimination and for which we had available data: body mass index (BMI) (Scott et al., 2008; Spahlholz et al., 2016) and life satisfaction (Assari & Caldwell, 2018; Fergusson et al., 2015).

To investigate the presence of, and adjust for, familial confounding in the studied association, we fitted within-and-between pair models, also using random effects and maximum likelihood estimation (Calais-Ferreira et al., 2022; Carlin et al., 2005). This approach fits the within-pair difference (the difference between the individual's value and the within-pair mean) and the within-pair mean separately for each risk factor in the model, allowing disaggregation of their shared (familial) and non-shared contributions to variance in perceived discrimination. When examined using a likelihood ratio test (LRT), if the within-pair difference and the within-pair mean differ statistically, there is evidence that the association is confounded by unmeasured factors, presumed to be familial.

We tested for interactions between the between-pair difference of each covariate and sex in separate models to obtain evidence of sex differences in familial confounding for each of these risk factors. We also present our regression analyses stratified by sex in the Supplementary Material.

We conducted sensitivity analyses, first restricting the regression analyses to those who experienced discrimination at wave 2 only (Supplementary Material, Table S1) and those who experienced discrimination in both waves 1 and 2 (Supplementary Material, Table S2). This was done to ensure a longitudinal relationship between mental disorder and perceived discrimination and to assess the precision of our primary outcome, respectively. Migrant status was a shared variable for all twin pairs; therefore, regression models including this variable were included in the sensitivity analysis (Supplementary Material, Table S3).

We fitted within-and-between models separately for male and female twin pairs and included interactive terms between sex and the between-pair difference of each covariate to assess sex differences in familial confounding (Supplementary Material, Table S4). We fitted a model restricting our definition of mental disorder to a diagnosis of anxiety or depression (Supplementary Material, Table S5) instead of the more heterogeneous mental disorder variable. We conducted ‘complete case' analyses for both within-individual and within-and-between pair models, whereby any individual or twin pair with missing data were excluded from the analysis. All analyses were conducted in Stata MP 16.0 (StataCorp, 2023). This study was reported according to the STROBE statement (von Elm et al., 2007).

### 2.4 Ethics statement

The authors assert that all procedures contributing to this work comply with the ethical standards of the relevant national and institutional committees on human experimentation and with the Helsinki Declaration of 1975, as revised in 2008. All procedures involving human subjects/patients were approved by the University of Melbourne's Human Research Ethics Committee (HREC ref#:21305). Ethics approval for the original TwinLife study has been obtained through the German Psychological Association (protocol numbers: RR 11.2009 and RR 09.2013). Written or verbal informed consent was obtained from all study participants.

## 3 Results

### 3.1 Descriptive statistics

Four participants were excluded for not having zygosity information. There were 1022 MZ and 1020 DZ same-sex pairs included in the analysis. Missing data were as follows: BMI (n = 454, 11%), current smoker status (n = 38, 1%), and global life satisfaction score (n = 76, 2%).

Table 1 provides descriptive statistics of the study sample. The mean age at baseline was 19.9 (Interquartile range = 17–23) years. There were 604 (14.8%) participants who self-reported discrimination at any wave. Around 8% (n = 332) of the sample reported having been previously diagnosed with a mental disorder at baseline, and no twins reported a new diagnosis between waves 1 and 2. Approximately 5% of the sample (n = 186) were migrants. Of those, 57 (31%) were recorded as being born in a country of the former Soviet Union, 28 (15%) from Eastern Europe and 26 (14%) from Arabic-Islamic countries.

**Table 1:** Descriptive characteristics of the study sample.

		Self-reported discrimination (n = 604)	No self-reported discrimination (n = 3480)	All (n = 4084)	*P*^a^
Diagnosed mental disorder, n (%)		90/604 (14.9)	242/3480 (7.0)	332/4084 (8.1)	<0.001
Male sex, n (%)		217/604 (35.9)	1511/3480 (43.4)	1728/4084 (42.3)	0.001
Age, years, mean (SD)		20.0 (3.1)	19.9 (3.1)	19.9 (3.1)	0.2
BMI, kg, mean (SD)		22.5 (4.3)	22.2 (3.9)	22.2 (3.9)	0.2
Current smoker, n (%)		150/601 (25.0)	840/3445 (24.4)	990/4046 (24.5)	0.8
Left school^b^, n (%)		17/604 (2.8)	65/3480 (1.9)	82/4084 (2.0)	0.1
Life satisfaction score^c^, mean (SD)		18.1 (4.5)	19.2 (4.0)	19.0 (4.1)	<0.001
Migrant, n (%)		61/604 (10.1)	125/3480 (3.6)	186/4084 (4.6)	<0.001

Note: All risk factors were measured at wave 1, except for ‘left school' which was defined as having left school at any wave.

a Two-tailed p-value of test for difference between those who did and did not self-report discrimination at waves 1 or 2. Chi-squared tests were used for binary variables and t-tests were used for continuous variables.

b Left school without obtaining a primary or secondary school qualification.

c Range: 5–25.

There was strong evidence for differences between groups with and without self-reported discrimination for mental disorder (*P*<0.001), sex (*P* = 0.001), global life satisfaction score (*P*<0.001), and migrant status (*P*<0.001).

### 3.2 Familial correlations

Sex-adjusted intra-class correlations (ICC) were 0.24 (95% Confidence Interval: 0.20–0.28) for mental disorder, 0.55 (95%CI: 0.51–0.58) for BMI, 0.49 (95%CI: 0.45–0.52) for current smoking status, 0.23 (95%CI: 0.19–0.27) for leaving school, and 0.36 (95%CI: 0.32–0.39) for global life satisfaction score. The sex-adjusted familial risk ratio for mental disorder was 6.54 (95%CI: 5.00–8.56, *P*<0.001).

### 3.3 Within-individual models

Table 2 describes the within-individual univariable and multivariable (adjusted) associations between mental disorder and other risk factors with experiencing discrimination. Having a mental disorder was positively associated with perceived discrimination in unadjusted and adjusted models, the latter with an OR of 2.24 (95%CI: 1.45–3.46, *P*<0.001). There was evidence of a negative association of male sex (aOR = 0.64, 95%CI: 0.48–0.85, *P* = 0.002) and life satisfaction (aOR = 0.93, 95%CI: 0.90–0.95, *P* < 0.001) with perceived discrimination in the univariable models. Migrant status was also strongly associated with perceived discrimination (OR = 4.97, 95%CI: 2.79–8.85, P<0.001).

**Table 2:** Within-individual unadjusted and adjusted associations between mental disorder and discrimination.

	n	OR (95% CI)	*P*
**Unadjusted**			
Diagnosed mental disorder	4084	2.79 (1.90–4.10)	<0.001
Male sex	4084	0.64 (0.48–0.85)	0.002
BMI, kg	3630	1.02 (0.99–1.06)	0.2
Life Satisfaction Score^a^	4008	0.93 (0.90–0.95)	<0.001
**Adjusted**^b^			
Diagnosed mental disorder	3571	2.24 (1.45–3.46)	<0.001

Note: All risk factors were measured at wave 1. OR (95% CI) = odds ratio with 95% Confidence Intervals.

a Range: 5-25.

b Model adjusted for sex, BMI, and life satisfaction.

### 3.4 Within-and-between pair models

Table 3 presents unadjusted and adjusted within-and-between pair associations between mental disorder and perceived discrimination. We did not find evidence of a within-pair association between mental disorder and perceived discrimination in unadjusted nor adjusted models, but found between-pair associations between diagnosed mental disorder (aOR = 6.04, 95%CI: 3.35–10.88, *P*<0.001), male sex (aOR = 0.64, 95%CI: 0.48–0.85, *P* = 0.002), and life satisfaction (aOR = 0.90, 95%CI: 0.87–0.94, *P*<0.001) with perceived discrimination, all in unadjusted models.

**Table 3:** Within-and-between pair unadjusted and adjusted associations between mental disorder and discrimination.

	n	Pair difference		Pair mean		LRT^a^
		OR (95% CI)	*P*	OR (95% CI)	*P*	*P*
**Unadjusted**						
Diagnosed mental disorder	4084	1.43 (0.85–2.42)	0.2	6.04 (3.35–10.88)	<0.001	<0.001
Male sex^b^	4084	–		0.64 (0.48–0.85)	0.002	–
Body-mass-index, kg	3630	1.01 (0.97–1.06)	0.5	1.03 (0.99–1.06)	0.179	0.6
Life satisfaction score^c^	4008	0.96 (0.92–1.00)	0.04	0.90 (0.87–0.94)	<0.001	0.02
**Adjusted**^d^						
Diagnosed mental disorder	3571	1.28 (0.71–2.30)	0.4	4.36 (2.24–8.49)	<0.001	0.01

Note: All risk factors were measured at wave 1. OR (95% CI) = odds ratio with 95% Confidence Intervals.

a Likelihood Ratio Test (LRT) statistic comparing within-and-between pair models with within-individual models.

b Only same-sex twin pairs were included in the study. Therefore, the estimates of male sex as a risk factor for discrimination did not have a paired difference, and within-individual and within-and-between pair models could not be compared.

c Range: 5–25.

d Adjusted for within-pair difference and within-pair mean of sex, body-mass-index, and life satisfaction score.

**Fig. 1:**
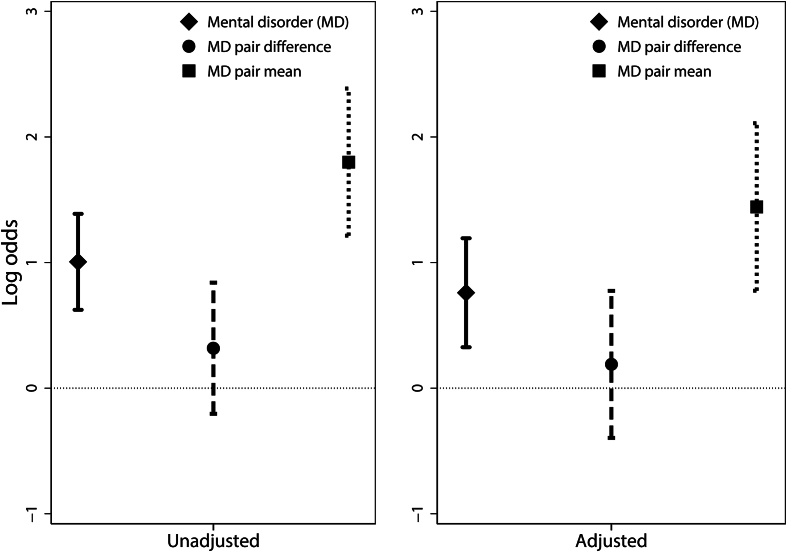
legend: Log odds of within-individual and within-and-between-pair associations between diagnosed mental disorder and experience of discrimination, including 95% confidence intervals.

Likelihood ratio tests (LRTs) indicated that the within-and-between pair models had a better fit than within-individual models only for mental disorder (*P*<0.001) in the unadjusted models. The fully adjusted within-and-between model offered a marginal improvement over the within-individual model (*P* = 0.01). Fig. 1 presents a visual comparison between within-individual (Table 2), and within and between-pair estimates (Table 3) of the association between mental disorder and perceived discrimination.

### 3.5 Sex differences

The sex differences analysis can be found in Table S5 (Supplementary Material). We found evidence of an interaction between male sex with the between-pair difference of mental disorder (*P* = 0.003). In the analysis stratified by sex, we found differences between the between-pair difference of mental disorder for males (aOR = 21.10, 95%CI: 5.82–76.56, *P*<0.001) and females (aOR = 2.15, 95%CI: 0.96–4.77, *P* = 0.06).

### 3.6 Sensitivity analyses

Our sensitivity analyses broadly supported our primary results. While the estimates in the sensitivity analyses were less precise due to studying only those who participated in both waves, we found no substantial differences in our main findings (Tables S1 and S2, Supplementary Material). Similarly, results from using mental illness (only anxiety and depression disorder) as an exposure (Table S4, Supplementary Material) did not produce considerably different results in the point estimates, although precision was again compromised.

## 4 Discussion

We investigated the association between mental disorder and perceived discrimination in young twin pairs in Germany using within-individual and within-and-between pair models, the latter to adjust for and assess the magnitude of familial confounding in this association. Our primary finding was that the within-individual association between having a mental disorder diagnosis and experiencing discrimination, which we observed, was largely explained by shared (paired), rather than individual, diagnoses of mental disorder. This was evidenced by the presence of an association between the within-pair mean but not the within-pair difference in mental disorder with perceived discrimination. We found formal evidence of familial confounding in the association between mental disorder and perceived discrimination.

Our results support the need for family-based mental healthcare models that incorporate family members of adolescents in the prevention and treatment of mental disorder, rather than exclusively focusing on individual behavioural factors. As mental disorder severity might be greater for individuals at higher genetic risk, incorporating a family history-informed approach to prevention and early intervention may improve both the efficiency and efficacy of these strategies (Kalman et al., 2022). This is supported by our evidence of co-twin's history of mental disorder being a major risk factor for having themselves a mental health diagnosis.

Societal efforts to address social inequalities that adversely affect families at higher risk of experiencing discrimination may be critical to achieving mental health equity. Thus, familial approaches to prevention should be complemented by universal interventions targeted at changing the determinants of stigma and discrimination more broadly (Henderson & Thornicroft, 2009). Nonetheless, mental health-related stigma and discrimination have been linked to a higher risk of unemployment, lower income, and higher healthcare costs (Sharac et al., 2010). Therefore, it is critical to interrupt this cycle of social disadvantage and mental ill-health early in life, prior to its entrenchment throughout the life course.

Another explanation for the results we observed is that having a co-twin experiencing mental health-related or general discrimination might heighten one's awareness of issues related to discrimination (due to mental health or other factors). This would result in more clustering of the reporting of such events in families and, therefore, reducing the number of pairs who are discordant for mental disorder or perceived discrimination. The familial clustering in this association, as well as a potential bi-directional association, present important methodological challenges that might be potentially addressed using other novel twin and family study designs (Davey et al., 2016), and including data from future waves of the TwinLife Study in longitudinal analyses.

Our study found that male twins from pairs where both were diagnosed with a mental disorder were at more than 21 (5.82–76.46) times higher odds of experiencing discrimination than male twins from unaffected pairs, compared to 2.15 (0.96–4.77) times higher odds for females, in the model adjusted for all covariates. This indicates that the familial confounding in the adjusted association between mental disorder and perceived discrimination differs by sex and that the risk of discrimination as a function of a family history of mental disorder is greater for males than females. The observed sex differences also have ramifications for interventions. The finding that familial confounding is more present for young males than females may indicate that males are more susceptible to the impact of shared environmental factors on mental health and perceived discrimination. Especially for males, family-based mental health interventions might better ameliorate the effects of discrimination. Understanding the familial determinants of adolescent and young adult mental health and perceived discrimination should be both an area for future research and a public health priority.

We observed that being a migrant was associated with perceived discrimination in the univariable within-individual model but not in the multivariable model, indicating individual-level confounding. Nonetheless, it is clear that migrants were disproportionally exposed to discrimination events in this cohort and prevention efforts targeted at young migrant youth may be warranted.

Our study findings support the concept of ‘intersectionality', in which perceived discrimination may be a result of multiple coalescing factors rather than just one cause (Armstrong et al., 2022), as it is likely determined by a complex network of interactions between genetic and environmental causes. This might also explain the well-known failure to reconcile estimates of (unmeasured) heritability of mental disorders and other health conditions found in twin studies with that observed in more recent genome-wide association studies with measured genetic variants (Feldman & Ramachandran, 2018). An approach to causal inference that considers genetic along with cultural and environmental differences within and between families within a wider set of social inequalities may yield better mental health and discrimination prevention strategies for adolescents and young adults. Studying any of these constructs while ignoring the other might represent a failure to achieve true equity and provide only temporary and insufficient answers to these major public health problems.

Our study had some limitations. The self-reported doctor's diagnosis of mental disorder, as well as not having available data on the diagnoses of more stigmatised disorders (such as psychotic disorders), might have under-detected the true prevalence of mental disorders in our sample. Any misclassification of this nature would result in a conservative measure of effect; therefore, this was unlikely to have considerably impaired this study. Of note, anxiety, depression, and ADHD, included in our study, are the three most prevalent mental disorders in German adolescents (Ravens-Sieberer et al., 2008). In any case, we note that our results are likely to be less generalisable to adolescents experiencing more severe mental health conditions, even if they follow a similar pattern of familial clustering observed in more common mental health conditions (Zavos et al., 2014). Further, our measure of self-reported discrimination was broad, and recall bias might also have influenced our estimates.

The inability to establish a direct timeline between the onset (or diagnosis) of mental disorders, the other risk factors included in our models, and perceived discrimination as the outcome limited potential causal inference in our study. There is a possibility that some of the covariates, such as BMI and life satisfaction, might lie on the causal pathway between mental disorder and perceived discrimination, and similarly for other risk factors studied in the adjusted models.

Our primary aim was to assess the hypothesis of familial confounding (i.e., confounding due to familial factors shared by twin pairs) in the association between perceived discrimination and mental disorders. Although our study had adequate power to address our primary aim, there is a residual possibility that we did not observe a true within-pair association between mental disorder and discrimination due to reduced statistical power. In our multivariable models, we additionally selected several covariates a priori as potential confounders because there was either prior empirical evidence or strong theoretical grounds for an association with both our exposure and outcome. However, as our aim was to adjust for these potential confounders, we did not account for multiple testing of these covariate associations. Therefore, caution should be applied when interpreting their corresponding associations with our outcome of interest as they may be prone to type II error and should be considered exploratory in nature. Our study might have been insufficiently powered to detect differences for multiple other covariates, as reflected by the relatively wide confidence intervals, which could demonstrate a lack of precision in these estimates. Future targeted examinations of the association between each of these covariates and mental disorders are warranted.

We intentionally grouped MZ and DZ pairs together in our regression analysis to increase the total sample size, precluding further disentangling genetic from shared environmental sources of confounding. Potential gene-environment correlation or interaction could not be tested in our models. Further research is needed to understand whether they may play a role in the association between mental disorder and perceived discrimination.

While the within-family study design here employed is an efficient tool to investigate associations holding familial (including genetic) factors constant, it can be less successful in fully considering differences between genetically and culturally diverse groups in our society. Importantly, the proportion of migrants was lower, and levels of education and income were slightly higher in the TwinLife study compared to the general German population (Mönkediek et al., 2019). Even considering that TwinLife used a population-based recruitment strategy, this indicates that our findings might not be directly generalisable to all contexts.

Our study is the first to provide evidence that the association between having a mental disorder and experiencing discrimination is confounded by unmeasured shared familial factors. Incorporating family members in interventions targeted at ameliorating mental ill-health and experiences of discrimination among adolescents may improve efficacy, especially for males.

## Funding

LCF is funded by a Suicide Prevention Australia Postdoctoral Fellowship. JTY receives salary and research support from a 10.13039/501100000925National Health and Medical Research Council Investigator Grant (GNT1178027). The TwinLife Project is funded by the German Research Foundation (grant number 220286500). Neither of the funders had any involvement in the preparation, study design, in collection, analysis and interpretation of data; in the writing of the report; nor in the decision to submit the article for publication.

## CRediT authorship contribution statement

**Lucas Calais-Ferreira:** Writing – review & editing, Writing – original draft, Project administration, Methodology, Formal analysis, Conceptualization. **Gregory Armstrong:** Writing – review & editing, Methodology. **Elisabeth Hahn:** Writing – review & editing, Methodology, Data curation. **Giles Newton-Howes:** Writing – review & editing. **James Foulds:** Writing – review & editing. **John L. Hopper:** Writing – review & editing, Supervision, Methodology. **Frank M. Spinath:** Writing – review & editing, Methodology, Data curation. **Paul Kurdyak:** Writing – review & editing. **Jesse T. Young:** Writing – review & editing, Methodology, Conceptualization.

## Declaration of competing interest

The authors have no interest to declare.

## Data Availability

Data will be made available on request.
