# Supplementary material for: Mental disorders and discrimination: A prospective cohort study of young twin pairs in Germany
Source: SSM Popul Health. 2024 Feb 7;25:101622. doi: 10.1016/j.ssmph.2024.101622 (PMC10877176; doi:10.1016/j.ssmph.2024.101622)
Supplement: Multimedia component 1 [file mmc1.docx]

**SUPPLEMENTARY MATERIAL**

| Table S1: Unadjusted and adjusted associations between mental disorders and other risk factors at wave 1 and discrimination (at wave 2) | | | | | |
| --- | --- | --- | --- | --- | --- |
|  |  | Univariable models | | Multivariable model | |
|  |  | OR | *p* | aOR | *P* |
| Diagnosed mental disorder |  | 2.96 (1.81–4.85) | <0.001 | 2.77 (1.57–4.90) | <0.001 |
| Male sex |  | 0.51 (0.34–0.75) | 0.001 | 0.51 (0.33–0.77) | 0.002 |
| BMI, kg |  | 1.01 (0.96–1.06) | 0.7 | 1.01 (0.97–1.06) | 0.6 |
| Satisfaction score |  | 0.93 (0.89–0.97) | <0.001 | 0.96 (0.92–1.00) | 0.06 |
| Note: this analysis was restricted to participants who participated in both waves of the study. See methods for details. | | | | | |

| Table S2: Unadjusted and adjusted associations between risk factors and discrimination (at both waves) | | | | | |
| --- | --- | --- | --- | --- | --- |
|  |  | Univariable models | | Multivariable model | |
|  |  | OR | *p* | aOR | *P* |
| Diagnosed mental disorder |  | 3.81 (1.81–8.01) | <0.001 | 3.77 (1.58–8.98) | 0.003 |
| Male sex |  | 0.55 (0.30–1.02) | 0.06 | 0.52 (0.26–1.04) | 0.07 |
| BMI, kg |  | 1.01 (0.94–1.09) | 0.7 | 1.02 (0.95–1.10) | 0.6 |
| Satisfaction score |  | 0.93 (0.87–0.99) | 0.02 | 0.96 (0.89–1.03) | 0.3 |
| Note: this analysis was restricted to participants who participated in both waves included in this study. See methods for details. | | | | | |

| Table S3: Unadjusted and adjusted associations between mental disorder and other risk factors with discrimination (at any wave), including migrant variable | | | | | |
| --- | --- | --- | --- | --- | --- |
|  |  | Univariable models | | Multivariable model | |
|  |  | OR | *p* | aOR | *P* |
| Diagnosed mental disorder |  | 2.79 (1.90–4.10) | <0.001 | 3.94 (1.76–8.84) | <0.001 |
| Male sex |  | 0.64 (0.48–0.85) | 0.002 | 0.49 (0.24–0.96) | 0.04 |
| BMI, kg |  | 1.02 (0.99–1.06) | 0.2 | 1.00 (0.94–1.08) | 0.9 |
| Satisfaction score |  | 0.93 (0.90–0.95) | <0.001 | 0.96 (0.90–1.03) | 0.2 |
| Migrant |  | 4.97 (2.79–8.85) | <0.001 | 2.89 (0.88–9.47) | 0.08 |

| Table S4: Unadjusted and adjusted associations between mental illness and other risk factors with discrimination (at any wave) | | | |
| --- | --- | --- | --- |
|  |  | Univariable models | |
|  |  | aOR | *p* |
| Diagnosed mental illness |  | 2.35 (1.44–3.83) | 0.001 |
| Male sex |  | 0.64 (0.48–0.85) | 0.002 |
| BMI, kg |  | 1.02 (0.99–1.06) | 0.2 |
| Satisfaction score |  | 0.93 (0.90–0.95) | <0.001 |
| Note: Mental illness was defined as diagnosed anxiety or depression. | | | |

| Table S5: Adjusted associations between within-pair difference and pair within-pair mean of risk factors (at wave 1) with experience of discrimination (at any wave) from within-and-between pair models, separately for males and females with an interactive term | | | | | | | | | | | | | |  | |
| --- | --- | --- | --- | --- | --- | --- | --- | --- | --- | --- | --- | --- | --- | --- | --- |
|  | Males | | | | |  | | Females | | | | | | Interaction with sex^1^ | |
| Risk factors | Pair difference | | Pair mean | | |  | | Pair difference | | | Pair mean | | |  |  |
|  | aOR | *P* | aOR | *P* |  | | aOR | | *P* | aOR | | *P* | P | |  |
| Diagnosed mental disorder | 1.23 (0.45–3.33) | 0.7 | 21.10 (5.82–76.56) | <0.001 |  | | 1.25 (0.60–2.58) | | 0.6 | 2.15 (0.96–4.77) | | 0.06 | 0.003 | |  |
| BMI, kg | 1.01 (0.93–1.09) | 0.9 | 1.05 (0.98–1.13) | 0.1 |  | | 1.02 (0.97–1.07) | | 0.5 | 1.02 (0.98–1.07) | | 0.3 | 0.4 | |  |
| Satisfaction score (X-Y) | 0.94 (0.87–1.01) | 0.09 | 0.93 (0.87–1.00) | 0.04 |  | | 0.98 (0.92–1.03) | | 0.4 | 0.93 (0.88–0.98) | | 0.009 | 0.7 | |  |
| ^1^ P-values of interactive term for sex and the pair mean of each risk factor. These estimates were obtained from a different model to those for males and females which were stratified and not adjusted by sex. | | | | | | | | | | | | | | | |
